# Microwhip Scorpions (Palpigradi) Feed on Heterotrophic Cyanobacteria in Slovak Caves – A Curiosity among Arachnida

**DOI:** 10.1371/journal.pone.0075989

**Published:** 2013-10-16

**Authors:** Jaroslav Smrž, Ĺubomír Kováč, Jaromír Mikeš, Alena Lukešová

**Affiliations:** 1 Department of Zoology, Faculty of Science, Charles University, Prague, Czech Republic; 2 Institute of Biology and Ecology, Faculty of Science, P.J. Šafarík University, Košice, Slovak Republic; 3 Institute of Soil Biology, Biology Centre of the Academy of Sciences of the Czech Republic, České Budějovice, Czech Republic; CNRS, France

## Abstract

To date, only morphological and anatomical descriptions of microwhip scorpions (Arachnida: Palpigradi) have been published. This very rare group is enigmatic not only in its relationships to other arachnids, but especially due to the fact that these animals dwell only underground (in caves, soil, and interstitial spaces). We observed the curious feeding habit of the microwhip scorpion *Eukoenenia spelaea* over the course of one year in Ardovská Cave, located in Slovakia's Karst region. We chose histology as our methodology in studying 17 specimens and based it upon Masson's triple staining, fluorescent light and confocal microscopy. Single-celled cyanobacteria (blue-green algae) were conspicuously predominant in the gut of all studied palpigrades. Digestibility of the consumed cyanobacteria was supported by the presence of guanine crystals, glycogen deposits and haemocytes inside the palpigrade body. Cyanobacteria, the oldest cellular organisms on Earth, are very resistant to severe conditions in caves, including even darkness. Therefore, the cyanobacteria are able to survive in dark caves as nearly heterotrophic organisms and are consumed by cave palpigrades. Such feeding habit is extraordinary within the almost wholly predacious orders of the class Arachnida, and particularly so due to the type of food observed.

## Introduction

*Eukoenenia spelaea* (**Peyerimhoff, 1902**) – microwhip scorpion (Arachnida: Palpigradi) –was first recorded in Slovakia at several caves [1], [2], although that group has a broader distribution in Europe and throughout the world. Most orders of the class Arachnida are made up of predators, including scorpions (Scorpiones), spiders (Araneae), whip scorpions (Thelyphonida), whip spiders (Amblypygi), solifuges (Solifugae), false scorpions (Pseudoscorpionida), harvestmen (Opiliones), and tickspiders (Ricinulei). Only in the mites order (Acarida) can many feeding exceptions be found (parasites, predators, phytophages), and there also exist several saprophagous harvestmen.

Small, poorly sclerotized and fragile, microwhip scorpions dwell in moist, stable habitats. In Europe, they have been found in caves and underground cavities generally [2], [3], [4], [5]. In the tropics, they inhabit also soil [4]. The genus *Leptokoenenia* dwells even in interstitial spaces [6]. Some authors have speculated they feed by predation, similar to other arachnids, such as by consuming arthropod eggs or small arthropods (e.g. mites) [7]. While there is no reported direct evidence of such feeding behaviour, the conspicuously large, chelate and scissors-like palpigrade chelicerae lend support to this view [8].

The rarity of palpigrades and difficulty in their sampling has not facilitated thorough study of their food selection. The internal microanatomy of the gut can nevertheless provide a useful vantage point for observing the feeding habit, and especially by examining the gut contents. Food actually consumed and digested can be visible there. Certain important contributions to our knowledge of that anatomy have been published [8], [9], [10], [11], and summaries of the internal anatomy have been compiled [4], [8]. The following microanatomical characteristics are summarized from the papers just cited:

The alimentary system consists of the mouth, pharynx, oesophagus, midgut (mesenteron or ventriculus) with its caeca, hindgut (rectum) and anus. The caeca, very usual parts of the alimentary tract in arachnids [3], [4], [9], [10], [11] are situated in prosoma (one pair) and in opisthosoma (three pairs). In or around the alimentary tract, free vacuolized cells have been reported with inclusions inside their vacuoles [9], [10]. The clusters of those cells are similar to the corpus adiposum of insects [12]. Moreover, concentric crystalline corpuscles have been recorded in the periphery of the alimentary tract. Those crystals were assumed to be metabolites or waste products, probably uric or guanine in nature [10] as birefringent corpuscles inside the body. The female reproductive system is comprised of two ovaries and two oviducts which converge in the uterus. The male reproductive system is comprised of paired testes. The structure of sperm and the spermatocytogenesis in *Prokoenenia wheeleri* have been recorded as special evolutionary derived characters [13]. That author pointed also to the isolated position of the palpigrades in relation to their sperm morphology [13].

The aim of this paper is to explain the feeding habit of microwhip scorpions in Slovakia's Ardovská Cave.

## Materials and Methods

Palpigrades were sampled at various sites within Ardovská Cave (south-eastern Slovakia, Domica area). The research adhered to the conditions of Licence # 3102/2009- 2.1/jam, from the Ministry of the Environment of the Slovak Republic, certificate of competency per Act No. 543/2002.

Histology was deemed the most appropriate method for such study. Ten specimens were fixed in modified Bouin-DuBosque-Brasil fluid [14]. Seven of these were embedded into Paraplast-plus (Sigma), sectioned on a Leica 2155 rotation microtome (0.005 mm thickness), then stained using Masson's triple stain. As a control, the same stain was applied only to plated cyanobacteria in a Petri dish. Three specimens were embedded into PolyBed/Araldit (Polysciences) epoxy resin and sectioned by diamond knife on an Ultracut (Reichert-Jung) (semi-thin sections of 0.0005 mm). All sections were observed under an AX-70 Provis (Olympus) light microscope, some of them using a Nomarski differential interference contrast prism. Some semi-thin sections were stained with acridine orange G (5% solution) (Sigma) and observed under fluorescent light for emission spectra exceeding 515 nm.

For scanning electron microscopy observations, the fixed (95.6% ethyl alcohol) animal was dehydrated using an increasing concentration of ethyl alcohol followed by a treatment with acetone and then plated with gold in a POLARON E 5100 sputter coater. A JEOL 6300 scanning electron microscope was used for photography.

Another three fresh, non-fixed animals were observed under a Leica TCS SP5 X confocal microscope with white light laser to create autofluorescence within the emission spectrum 498–627 nm.

## Results

**Figure 1:**
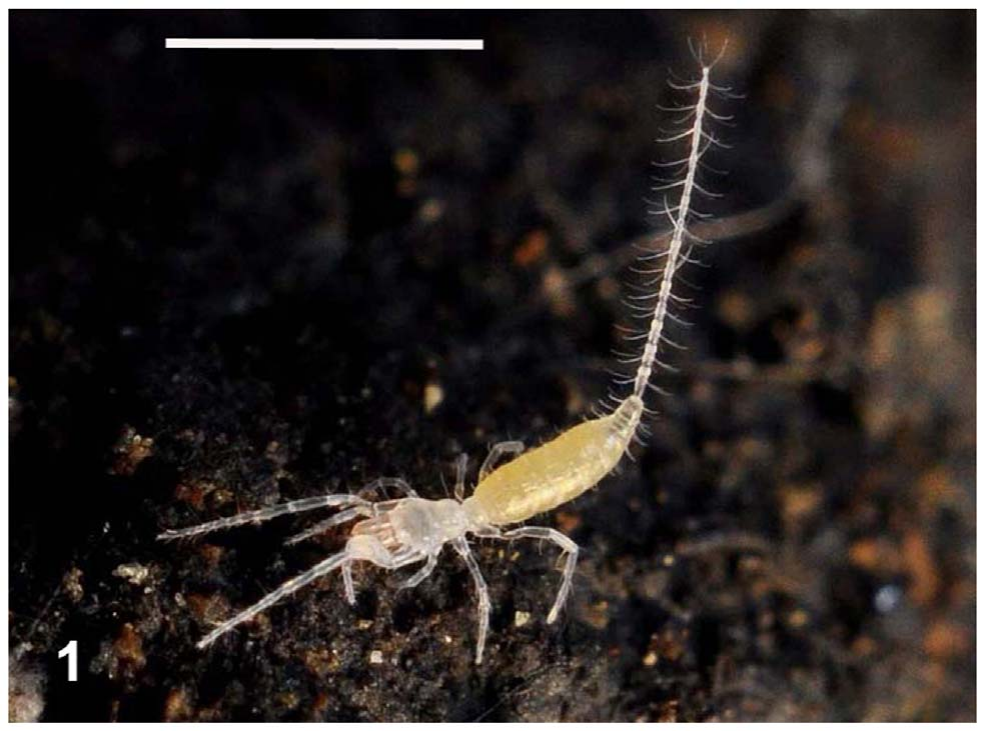
Live *Eukoenenia spelaea* in its cave habitat. Scale bar  = 1 mm.

**Figure 2:**
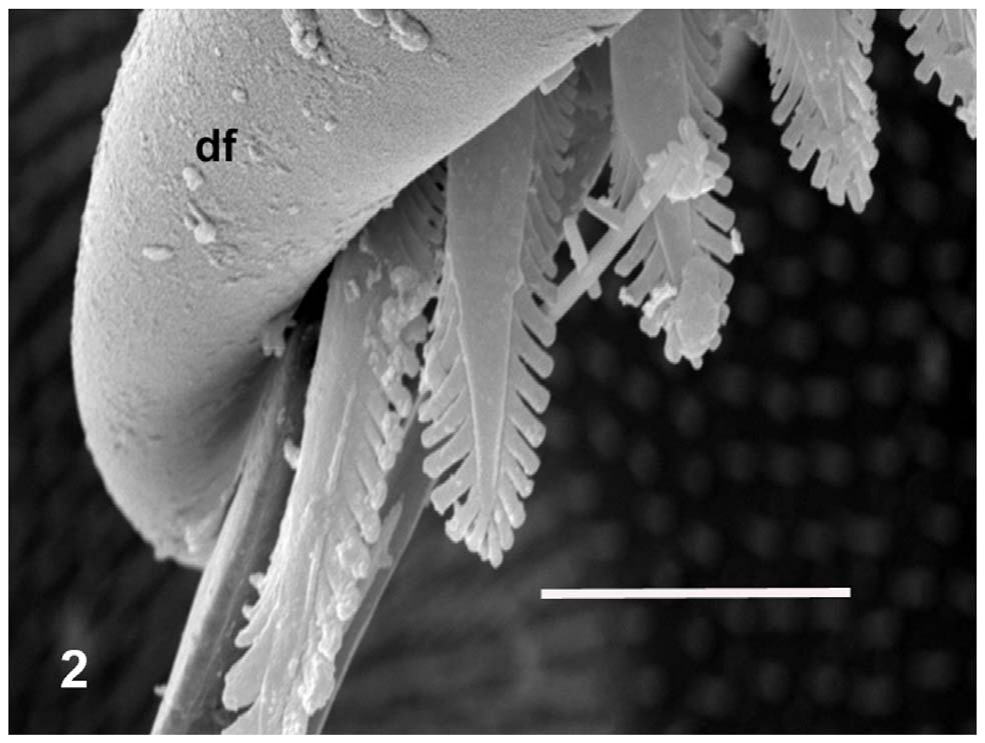
Chelicera with fringes under scanning electron microscopy. Abbreviation used: df =  the fixed digit of the chelicera. Scale bar  = 0.01 mm.

**Figure 3:**
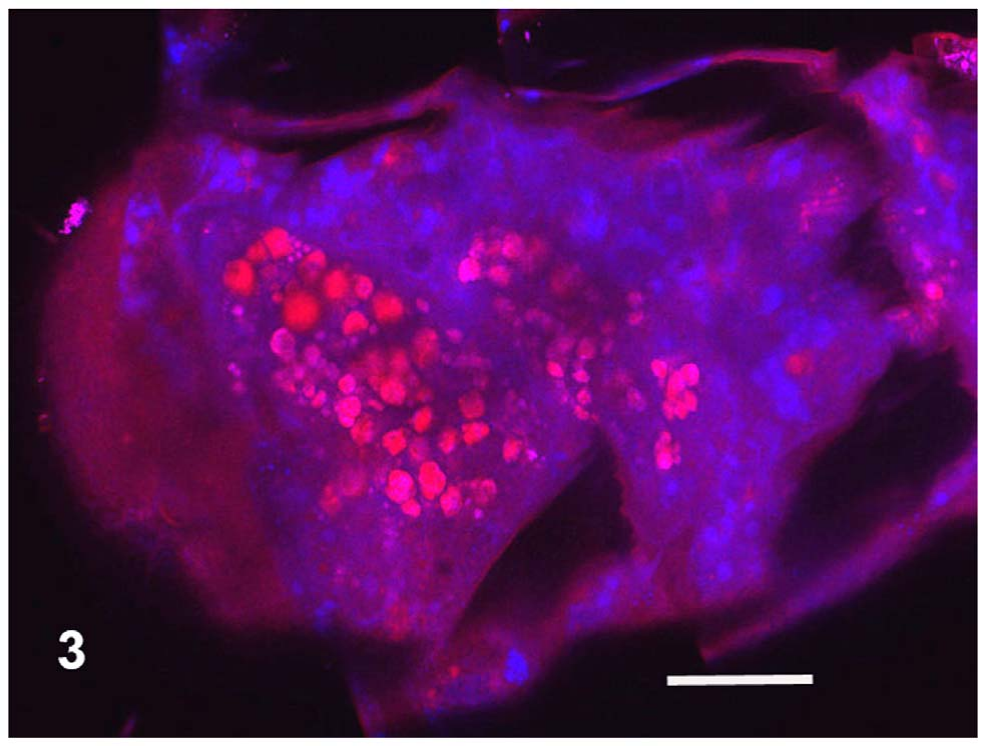
Part of the intact animal. Confocal microscopy, autofluorescence within the emission spectrum exceeding 498–627 nm. Red, roughly circular spots are cyanobacteria. Pink blotches are glycogen. Scale bar  = 0.02 mm.

The body colouring of the fresh, intact specimens of microwhip scorpions was yellowish (Figure 1). Long, three-segmented chelicerae are situated before the mouth of microwhip scorpions. They are chelate, with the appearance of a scissors-, forceps- or pincers-like tool. The cheliceral teeth appeared to be of a setal nature under light microscopy, but the scanning electron microscope revealed their more sophisticated form. Those flat teeth were equipped at the margin with rather fringe-like smaller teeth, thus creating a comb-like appearance (Figure 2). Observing autofluorescence of fresh non-fixed individuals under the confocal microscope revealed larger, red cells inside their bodies (Figure 3).

**Figure 4:**
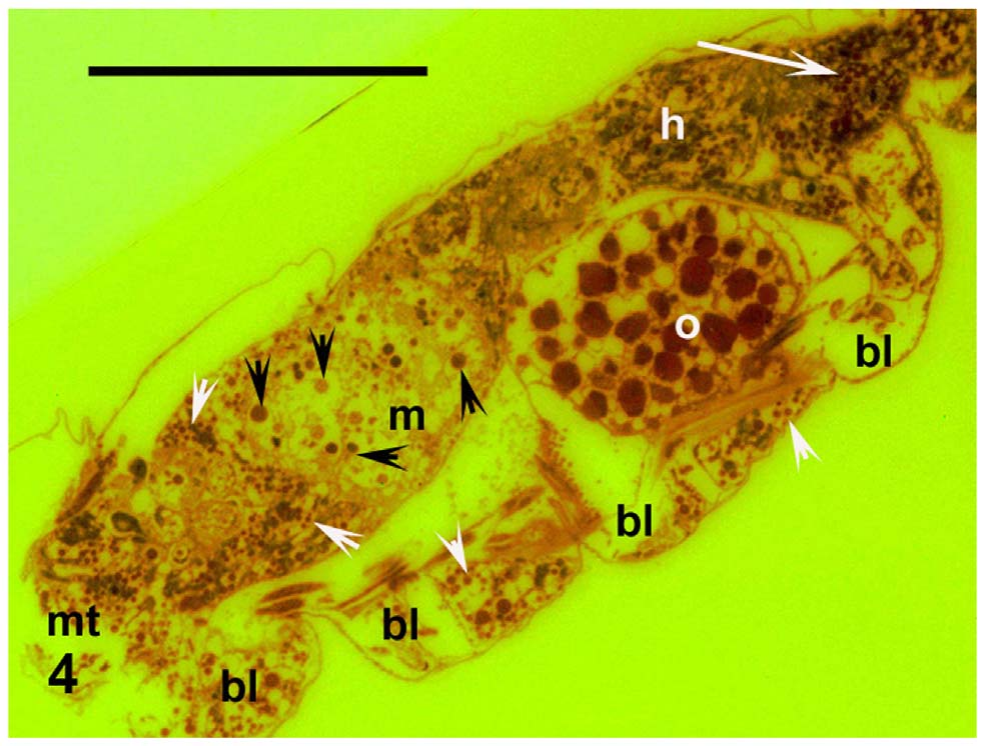
The entire animal. Cyanobacterial cells in midgut (black arrowheads), mixture of concentrated cells and amorphous organic matter in hindgut (white arrow), and glycogen deposits in periphery (diverticula) of gut (white arrowheads), sagittal section. Fluorescence with emission spectra exceeding 515 nm, colours inverted, orange G staining. Abbreviations used: bl =  diverticula of the gut in bases of the legs, h =  hindgut, m =  mesenteron or midgut, mt =  mouth area, o =  egg. Scale bar  = 0.1 mm.

**Figure 5:**
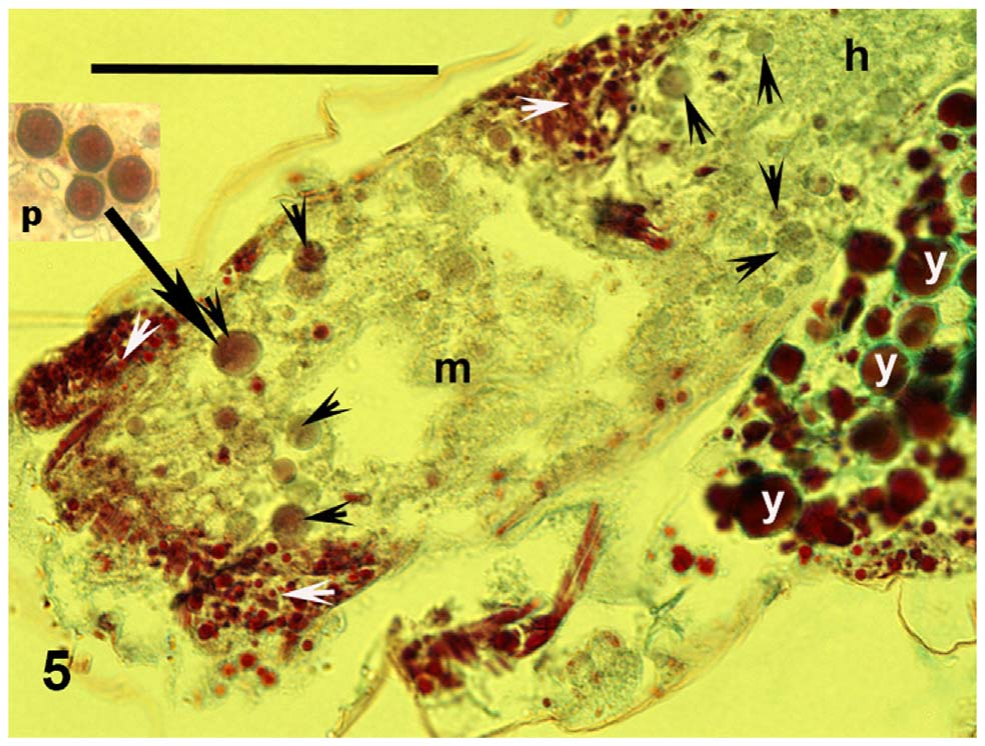
Midgut. Cyanobacterial cells (black arrowheads) and glycogen deposits (white arrowheads), black arrows points from plated cyanobacteria (rectangle out of body) to consumed cell in midgut. Parasagittal section. Masson's trichrome. Abbreviations used h =  hindgut, m =  mesenteron or midgut, p =  plated cyanobacteria, y =  yolk granules. Scale bar  = 0.05 mm.

**Figure 6:**
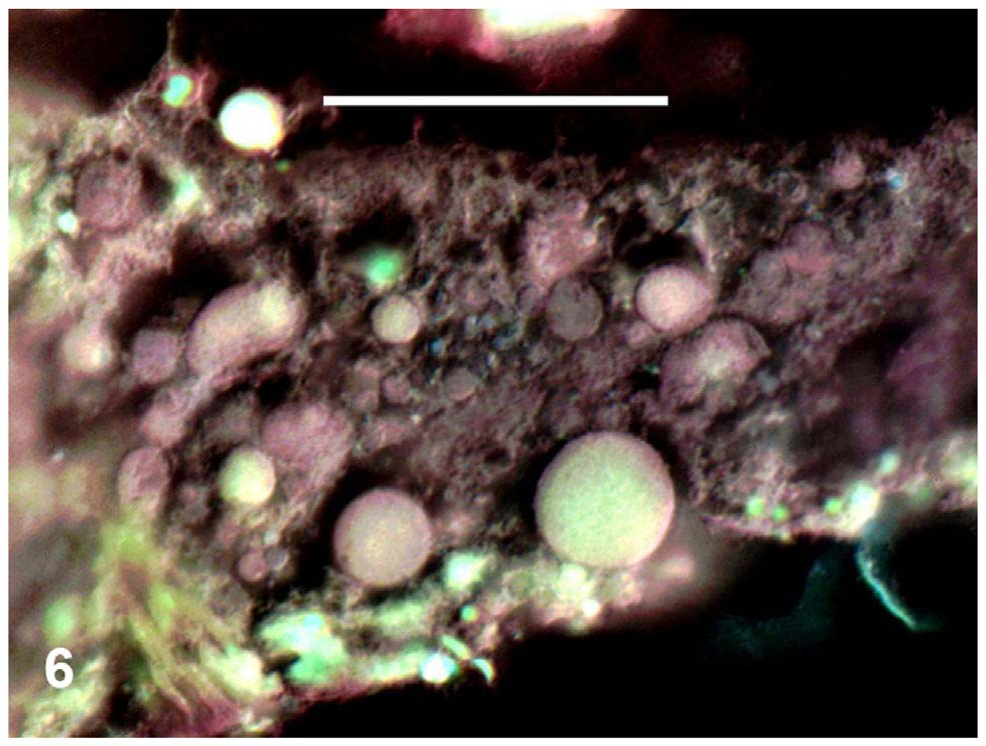
Detail of midgut crowded by cyanobacterial cells. Parasagittal section. Masson's trichrome combined with Nomarski differential interference contrast microscopy, with colours inverted. Scale bar  = 0.02 mm.

The midgut (mesenteron or ventriculus) is the largest part of the alimentary system (Figures 4 and 5). It projects into lateral diverticula extending into the bases of the legs. The mesenteric cavity was mostly filled or quite crowded with spherical cells of varying size. These ranged from 0.003 mm, but most were around 0.006–0.008 mm or larger (Figures 4, 5 and 6). These cells were stained violet or pinkish by Masson's trichrome, although some were only greenish (Figure 5). Their surfaces were smooth. These cells contained no nuclei, but very small vesicles looking like vacuoles were visible within them (Figure 5). The Masson's staining resulted in the same colour as was visible for single-celled cyanobacteria (*Chroococcidiopsis*) plated simultaneously on medium and stained on a microslide (cf. “p” in Figure 5). Moreover, the smaller, bacterial cells were scattered in the mesenteron between the described larger cells. In the following hindgut, there proceeded a concentration of the gut contents (Figure 4). While many cells in the mesenteron were violet or pinkish, in the hindgut most of the large spherical cells were greenish (Figure 5). In addition to those large cells, small dark as well as green particles were mixed with amorphous matter in the hindgut lumen (Figures 4 and 5).

**Figure 7:**
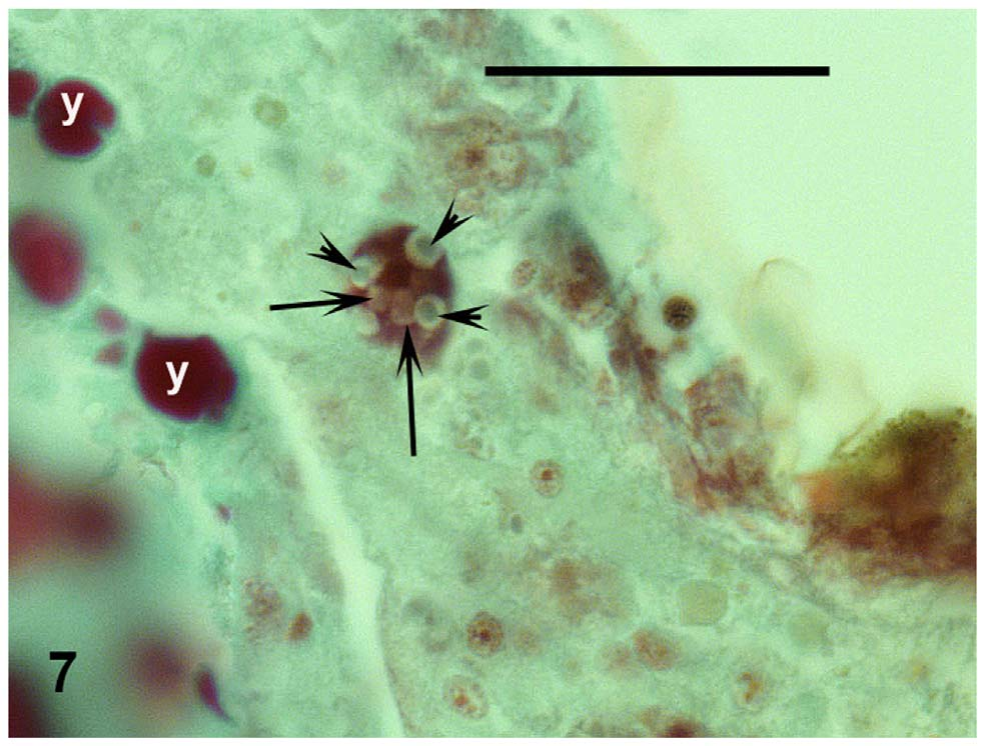
Haemocyte in midgut with guanine crystals (arrowheads) and other substances in vacuoles (arrows). Masson's trichrome. Abbreviation used: y =  yolk granules. Scale bar  = 0.02 mm.

Moreover, another type of cells was visible in the gut. Their size was around 0.006–0.007 mm. These were conspicuously vacuolized and stained intensively red. Greenish crystalline or grey matter was observed in their voluminous vacuoles (Figure 7).

Finally, very intensively red, small, spherical corpuscles, 0.002–0.003 mm in diameter and without nuclei filled the periphery of the alimentary tract, including the tops of the mentioned diverticula (Figures 4 and 5).

All the analysed individuals were females, each with one matured egg (Figures 4 and 5) that was non-nucleated, around 0.006 mm in diameter, and filled with yolk granules.

## Discussion

### Cyanobacteria as digestible food

The food offer in caves seems to be poor. Predation is one possible feeding habit, and this is very usual among most arachnids. The long chelicerae of palpigrades make them look predacious, and, with their three segments, these are evolutionarily very ancient [8], [15]. But their flat teeth are fringed or flanked by small marginal teeth. Hence, the organ as a whole looks more like a comb or brush than the forceps of a predator. The midgut (mesenteron) of the palpigrades contained mostly the above-described separated spherical cells of varying size without nucleus and with small vesicles inside. Those cells strongly resembled single-celled cyanobacteria (frequently of size exceeding 0.006–0.007 mm). Red light emission under the confocal microscope was characteristic of single-celled cyanobacteria. Cyanobacteria are probably the oldest cellular organisms on Earth [16], [17], [18]. The oldest known fossils, in fact, are cyanobacteria from Archaean rock of western Australia, dated as 3.5 billion years old [18], [19]. During that very long time, cyanobacteria have sustained themselves and refined their survival mechanisms under extreme conditions—in deserts, in the Antarctic region, under and within rocks, as well as in caves [20]. They are very resistant against low oxygen levels, very high or very low temperature, and poor light [21], [22]. The light conditions in caves force cyanobacteria to survive under the very poorly photoautotrophic conditions of nutrition occurring there [21], [23], [24], even though most of the cyanobacteria are considered to be clearly photoautotrophic organisms. In addition to autochthonous cyanobacteria, some can be floated into the cave from its external surroundings. Moreover, cyanobacteria have exhibited a very strong phenoplasticity, hence, a variability of characteristics under pressure of environmental factors. Indeed, cyanobacteria have been found in several caves under complete darkness [24], [25], [26]. The cyanobacteria from palpigrades gut in Ardovská Cave are single-celled and very similar to the order Chroococcales. That group of cyanobacteria has been found also in caves in, for example, Greece [25], Spain [26] or Russia [27]. These organisms exhibit extremely high resistance against abiotic factors, including darkness, as confirmed at Israel's Timna National Park [20] where the genus *Chroococcidiopsis* (Chroococcales) dwells in the Nubian sandstones as a cryptoendolithic organism, hence, inside such rocks [20]. The cyanobacterial photoautotrophy seems to be restricted [21]. Even while morphological stability of cyanobacteria through the long evolutionary period has been assumed, they nevertheless exhibit conspicuous genetic changes under fluctuating biogeochemical conditions. These have contributed to their adaptability due to genetic transfer and changes in DNA [16].

### Support of digestibility of cyanobacteria

In our microwhip scorpions, moreover, other types of cells were found apart from cells consumed as food. These were conspicuously vacuolized, stained intensively red, and appeared similar to cells found previously in mesentera of palpigrades [9]. Such free cells, haemocytes, are able to transport nutrients or wastes, including guanine, in arthropods [28], [29], [30]. The greenish crystals in the vacuoles in palpigrades appeared to be guanine, the universal arachnid waste substance [31]. This indicates digestion of a food rich in nitrogen [32]. Guanine crystals were reported by Millot also in palpigrades [10]. The tops of gut diverticula were filled by red granules corresponding, according to their staining, to glycogen particles – the usual nutritional storage product in many arachnids [33]. Glycogen also has been commonly reported in cyanobacteria (so-called cyanobacterial starch, α-1-4 glucan) [21]. Those energy-rich deposits indicate the consumption of a substantial amount of food digestible [32] for palpigrades, as confirmed by the red-stained deposits in the periphery of the alimentary tract in our microwhip scorpions as well as in Millot's animals [10].

### Extraordinary position of palpigrades

In addition to predators, as well as necrophagous, omnivorous, saprophagous and bacteriophagous animals in caves [34], [35], microwhip scorpions (Palpigradi) can be considered feeding specialists for autochthonous or allochthonous cyanobacteria as digestible food, at least in Slovakia's Ardovská Cave. The guanine crystals in haemocytes and glycogen deposits support the digestibility of cyanobacteria for palpigrades. This non-predacious food selection represents an unexpected and extraordinary feeding specialization among the mostly predacious arachnids.
